# Study on Stability and Applicability of Calcium Carbide Slag—Dealkalized Red Mud and Solid Waste Composite Materials in Road Materials

**DOI:** 10.3390/ma18133140

**Published:** 2025-07-02

**Authors:** Wentong Wang, Shuqian Wang, Yu Cheng, Meng Jia, Jianyang Gao

**Affiliations:** 1 College of Transportation, Shandong University of Science and Technology, Qingdao 266590, China; wwt@sdust.edu.cn (W.W.); wsq15376118551@outlook.com (S.W.); skd996718@sdust.edu.cn (Y.C.); 2 CHALCO Shandong Co. Ltd., Zibo 255052, China

**Keywords:** red mud, CT scan, unconfined compressive strength, solidification, industrial solid waste

## Abstract

The storage of highly alkaline red mud (RM) consumes land and threatens the environment, making its reuse crucial. The study used calcium carbide slag to dealkalize it, and analyzed the changes in mineral particles in RM using a CT scan. It then evaluated the stabilization effects of different materials and explored the mechanism of RM solidification through analysis of micro-mechanisms. The results showed that after dealkalization with CCS, RM particles form more agglomerates and the overall structure becomes more compact, and the Na^+^ content in RM decreased from 10.44 wt% to 0.86 wt%. After treatment with stabilization materials, the mechanical strength of low-alkalinity RM was greatly improved, and the stabilization effect of composite slag was the best. When the partial replacement ratio was 12%, the 28 d compressive strength was 4.51 MPa. After soaking in water for one day and night, the strength decreased by 24.3%, which had good stability. This study found that the strength gains were mainly due to crystal substances like Ca_3_Al_2_O_6_ and non-crystalline substances such as C-S-H filling pores and wrapping particles. This study provides a new method for RM stabilization and promotes the utilization of industrial by-products.

## 1. Introduction

Red mud (RM) is a high alkaline solid waste after extracting alumina from bauxite, and the output of each ton of alumina is about 1.0~2.0 t RM [1,2,3]. With a global stockpile exceeding 4 billion tons and annual emissions surpassing 120 million tons, it is predominantly stored in disposal areas near alumina production sites through stacking or landfilling [4,5,6]. This not only wastes land resources, but its high alkalinity also poses a serious risk of soil and water pollution, thereby limiting the sustainable development of the aluminum industry [7,8,9]. Therefore, the reuse of RM has become the focus of current research.

Currently, the main ways of RM recovery are the preparation of building materials [10], the extraction of valuable metals, and the development of environmental remediation materials [11]. However, due to its special physical properties, environmental risks, and economic costs, these methods are still in the early stage, and the utilization rate of RM is low. Application in road materials is considered one of the most effective ways for large-scale, harmless, and resourceful utilization of RM. But if RM is directly used as a road base material, it will cause soil pollution, and the free alkali in RM will migrate to the surface, causing structural cracking and weathering, thereby reducing material strength and durability and failing to meet usage requirements. Furthermore, it has been found that after dealkalization treatment, the California bearing ratio of RM is improved [12], which is beneficial for subsequent stabilization treatment or application of RM. Therefore, dealkalization is a prerequisite for the further comprehensive utilization of RM [13]. Calcium carbide slag (CCS), a waste from calcium carbide production, occupies lots of land for disposal [14]. Its high CaO content worsens environmental pollution and causes resource waste. However, this high CaO level allows it to replace natural calcium-based materials and be used for RM dealkalization [15]. The calcium ions can react with alkaline substances in RM to form tricalcium aluminate, replacing Na^+^, thereby reducing the alkalinity of RM [16,17]. Wang et al. used CCS to dealkalize RM and obtained that the Na_2_O content in RM was less than 3%, indicating that the dealkylation effect was good [18]. Huang et al. found that under optimal conditions, dealkalized RM from CCS had residual Na_2_O and K_2_O reduced to below 0.93% and 0.45%, respectively [19]. These studies indicate that CCS is technically feasible as an RM dealkalizing agent. Utilizing the principle of treating waste with waste, the alkalinity of RM can be adjusted through the application of industrial waste. However, the microstructural evolution of RM during dealkalization remains underexplored. To address the issues of low particle size, poor stability, and low strength of RM, it was found that adding stabilizers can improve its strength and stability [20,21]. Tan et al. [11] found that mixing RM and cement (C) at a 1:1 ratio greatly improves the material’s early strength. Also, adding 3% to 9% C to RM boosts its UCS, which increases with more C. The mechanical properties of materials can also be improved by adding chromium slag and blast furnace slag (GBFS) to RM. Cen et al. [22] used C to replace 10%~90% of RM. The results showed that with the increase in C content, the flexural and compressive strength of the paste increased. At the same time, some studies have improved the overall strength of materials by adding additional activators. Hu et al. [23] used low-concentration NaOH as an alkali activator and mixed fly ash and RM at a ratio of 5:5 to prepare cementitious material with a compressive strength of 15.2 MPa. Zhong et al. [24] mixed GBFS and RM at a 7:3 ratio and added Na_2_SiO_3_ as an activator, thereby synthesizing geopolymers with a compressive strength of 72.19 MPa. Some researchers have used RM and GBFS as precursors and sodium silicate and sodium hydroxide as composite activators, but the overall strength of the material was still low. Due to the high cost of preparation, these methods are rarely used on a large scale. Currently, the use of industrial waste residues for the solidification treatment of RM has become a hot topic of research, while studies on the solidification effects of different waste materials on low-alkalinity RM are relatively scarce. In most studies, the amount of RM used is limited to ensure the material meets overall strength requirements, thereby failing to achieve the goal of extensively utilizing RM. Therefore, it is imperative to select one or more solidification materials with relatively optimal solidification effects to enhance the quantity of RM utilized.

Studies have shown that the comprehensive utilization of multiple solid wastes can effectively improve material properties while solving solid waste disposal problems, maximizing the use of resources, and achieving sustainable development [25,26,27]. M. Jothilingam et al. [28] mixed FA, RM, and GBFS, and used NaOH and Na_2_SiO_3_ as alkaline activators to make polymer concrete. Wang et al. [29] used a CCS, RM, and FA ternary binder system and added municipal solid waste incineration fly ash (MSWIFA) to prepare samples at a ratio of 3:3:2:2. The 28 d compressive strength reached 11.6 MPa. This approach co-activated CCS and MSWIFA, improving the overall performance of the composite material. Gao et al. [30] produced sulphoaluminate C using RM, GBFS, CCS, steel slag, and flue gas desulphurization gypsum based on synergistic theory and were able to achieve a 28d compressive strength of 29.3 MPa, proving that there is a synergistic effect between the raw materials.

This study aims to investigate changes in mineral particles within RM before and after dealkalization, and the solidification effects of different materials on low-alkalinity RM. It also explores the solidification mechanisms of various materials in solidifying low-alkalinity RM. First, the changes in the mineral composition characteristics of red mud before and after dealkalization were analyzed through CT scanning. Then, in order to study the stabilization effects of different materials on low-alkalinity RM, the experiment selected four stabilization materials (cement, composite slag, titanium gypsum, and fly ash) and studied their influence on the compaction performance, mechanical strength, and water stability of the materials in terms of their admixture dosage and curing time. Finally, the stabilized mechanism of low-alkalinity RM was explored, and the microstructure and chemical composition were investigated using X-ray diffraction and scanning electron microscopy. The research results provide support for the effectiveness of using CCS to treat RM for dealkalization and provide a reference for selecting suitable RM solidification materials, enabling further understanding of the solidification mechanism of low-alkalinity RM and exploring the application potential of RM in road engineering.

## 2. Materials and Methods

### 2.1. Materials

#### 2.1.1. Red Mud and Calcium Carbide Slag

The RM and CCS used in this study were supplied by China Aluminum Shandong Co., Ltd. (Zibo, China). The main chemical components of the raw materials were determined by X-ray fluorescence (XRF). Rapid qualitative and quantitative analysis of elements from 6C to 92U with an elemental detection range of 0.0001–100% was conducted using a fully automated scanning X-ray fluorescence spectrometer (ZSX Primus II) from Rigaku, Tokyo, Japan, to test the chemical composition of the raw materials used [31]. The analysis results are shown in Table 1. XRF analysis showed that the main chemical compounds in RM were Fe_2_O_3_, Al_2_O_3_, Na_2_O, and SiO_2_, while CCS mainly contained CaO. In addition, the particle size distribution of RM and CCS was tested by a laser particle size analyzer, and the results are shown in Table 2.

**Figure 1:**
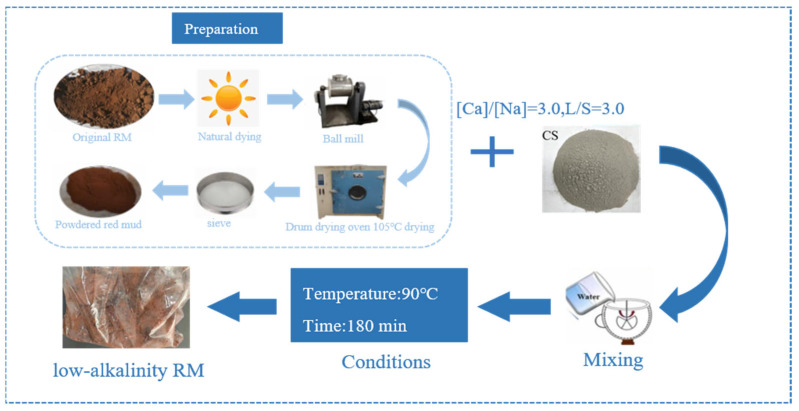
Process of dealkalization of RM with CCS.

The alkali in RM, apart from some attached alkali, mainly exists as combined alkali in the form of sodium silicate slag (Na_2_O·Al_2_O_3_·nSiO_2_·mH_2_O). By converting insoluble sodium ions in RM into soluble sodium ions, the Na_2_O in sodium silicate slag is converted into calcium silicate slag (3CaO·Al_2_O_3_·nSiO_2_·(6 − 2n)H_2_O). The Na_2_O then enters the solution and can be completely leached out by washing with water, thereby achieving the goal of alkali removal from RM [32]. From the XRF results, it can be seen that CCS has a high content of CaO (62.6%), which can be utilized to achieve RM dealkalization by utilizing Ca^2+^ in CCS to react with alkaline minerals in a substitution reaction, which results in the conversion of Na into Na^+^ to be dissolved and then removed by washing. The RM was reacted with CCS for 180 min according to the calcium to sodium ratio, i.e., [Ca]/[Na] = 3.0, at L/S = 3.0 and a temperature of 90 °C to obtain a low alkaline RM. The specific steps for the treatment of RM by dealkalization using CCS are shown in Figure 1.

#### 2.1.2. Stabilization Materials

The stabilization materials used in this study are cement (C), composite slag (CS), titanium gypsum (TG), and fly ash (FA), of which C and FA are from Chalco Shandong Co. Ltd. (Zibo, China), CS is from Shandong Iron and Steel Group (Jinan, China), and TG is from Shandong Dongjia Titanium Dioxide Factory (Zibo, China). The main chemical compositions of each material are analyzed by XRF, and the results are shown in Table 3, showing that the main chemical complexes are CaO and SiO_2_ in C, CaO, and SiO_2_, and Al_2_O_3_ in CS, SO_2_, CaO and SiO_2_ in TG, while the main chemical composition is Fe_2_O_3_ in FA.

The cement used in the test was tested in accordance with the GB/T 17671-2021 “Test Method of Cement Mortar Strength (ISO Method)” standard [33] to ensure that it met the specified strength grade requirements, thereby ensuring the reliability of the subsequent test results. The cement strength index obtained is shown in Table 4.

The particle size index of CS was determined, and the results are shown in Table 5 [34]. From Table 5 particle size index, it can be seen that CS particles are all fine and uniformly distributed, which can improve the workability and strength of the material when it is used as an admixture.

The FA was tested for the loss on ignition, moisture content, and particle size distribution, and the results are shown in Table 6. From the sieve passage rate, it can be seen that the FA particles are fine, and it has the ability to improve the denseness and strength of the material and improve the durability of the mixture. Its water content is 0.69%, which maintains a good dry state and ensures the stability and activity of FA in use.

### 2.2. Sample Preparation

In order to investigate the effect of stabilization material type and partial replacement ratio amount on the stabilized effect of low-alkalinity RM, four materials are selected for the test, and seven ratios are set up to increase uniformly from 0% to 12% at 2% intervals. The specific partial replacement ratio and number are shown in Table 7. Among them, the group with 0% admixture is defined as the control group.

According to the design ratios, low-alkalinity RM, water, C, CS, TG, and FA were poured into the mortar tank and mixed thoroughly. The homogeneous mixture was then poured into 50 mm × 50 mm cylindrical molds and compacted using a standard heavy-duty compaction apparatus. Finally, the specimens were placed in a standard curing room (temperature 20 ± 2 °C, humidity ≥ 95%) for curing periods of 3 d, 7 d, and 28 d. To ensure the reliability of the test data, three parallel tests were set up for each group of stabilization material and partial replacement ratios.

### 2.3. Test Methods

#### 2.3.1. Compaction and Stability Tests

According to JTG 3441-2024 [34] “Test Procedure for Inorganic Binding Material Stabilization Materials for Highway Engineering”, the optimum moisture content (OMC) and maximum dry density (MDD) of the specimens with different dosages were tested for compaction. Unconfined compressive strength (UCS) tests were carried out on the specimens uncured and cured for 3 d, 7 d, and 28 d, and the UCS values of the specimens were tested again after soaking for one day and one night to ensure the water stability of the materials [34].

#### 2.3.2. Microscopic and Morphological Testing

(1) X-ray diffraction (XRD)

The specimens were ground to a particle size of 320 mesh and scanned and analyzed with Cu radiation in steps of 0.0001°/step from 10° to 90° (2-theta) using an EMPYREANX-ray diffractometer from PANalytical, Almelo, The Netherlands, with an operating current of 60 mA and an operating voltage of 60 kV.

(2) Scanning electron microscopy (SEM)

SEM analysis (2000×, 5000×, and 20,000×) was performed on the crushed samples to characterize the microstructure of the hydration products under the microscope, and energy-dispersive spectrometry (EDS) analysis was performed on the RM before and after dealkalization to study the changes in elemental composition.

(3) CT image acquisition and processing

**Figure 2:**
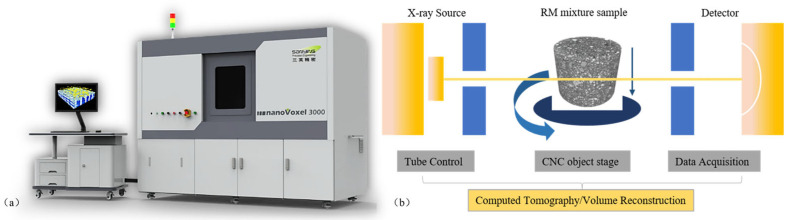
(**a**) CT scanner; (**b**) working principle.

**Figure 3:**
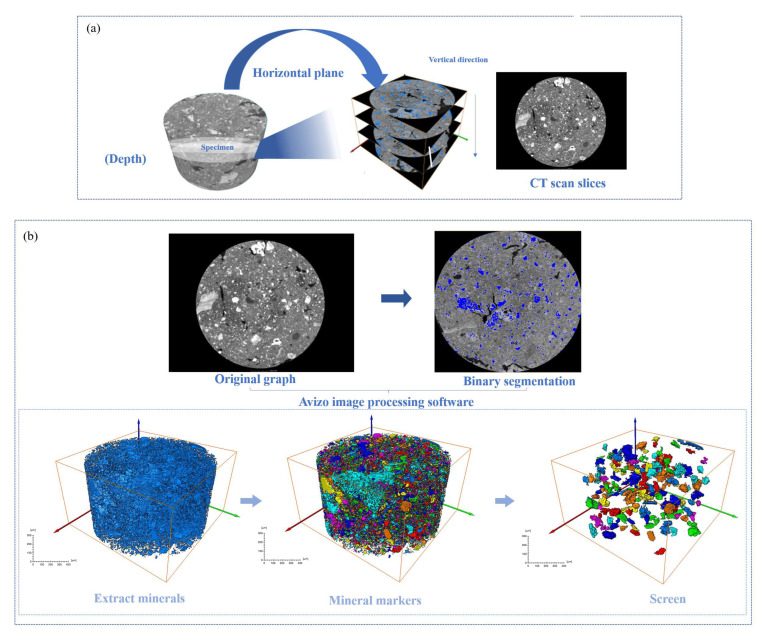
Image segmentation effect diagram: (**a**) image preprocessing; (**b**) 3D reconstruction.

Images of the internal structure of the RM were obtained by X-ray computed tomography (CT) scanning. The CT scan used in this study was a nanoVoxel-3000 series open-tube transmission high-resolution system (Sanying Precision Instruments Co., Ltd., Tianjian, China), and a picture of the scanner and its working schematic are shown in Figure 2. Radial and axial cross-section images with a resolution of 0.5 μm were generated using a scanning voltage of 60 kV and a current of 30 μA. The obtained CT images were thresholded and segmented, and Figure 3a shows an example of radial images obtained from the CT scan [35]. In these images, the white areas represent RM aggregates and the black parts represent voids and exteriors.

**Figure 4:**
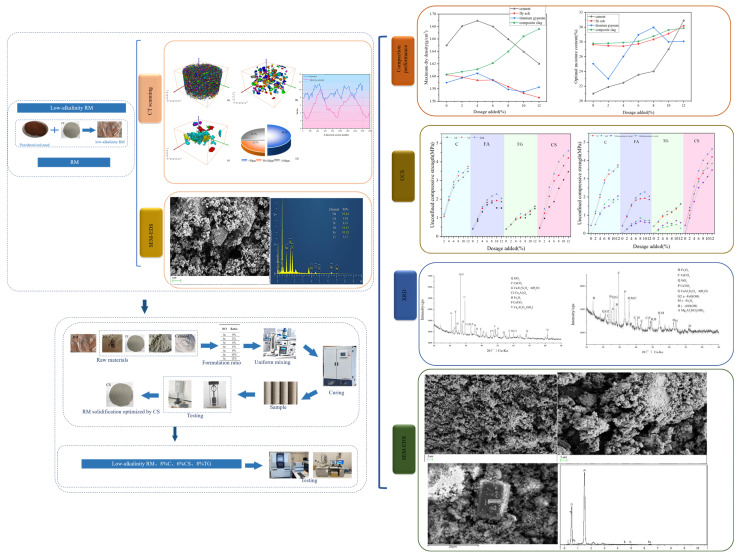
The research process.

The 3D CT scan images of the specimens were reconstructed using Avizo 3D reconstruction software (https://cases.amira-avizo.com/use-cases?search=%223D%20reconstruction%22), and the extracted minerals were marked with different colors [36]. The 3D reconstruction process of the specimen is shown in Figure 3b. The specific research process is shown in Figure 4.

## 3. Results and Discussion

### 3.1. CT Analysis

#### 3.1.1. Analysis of RM Mineral Particle Distribution Before and After Dealkalization

**Figure 5:**
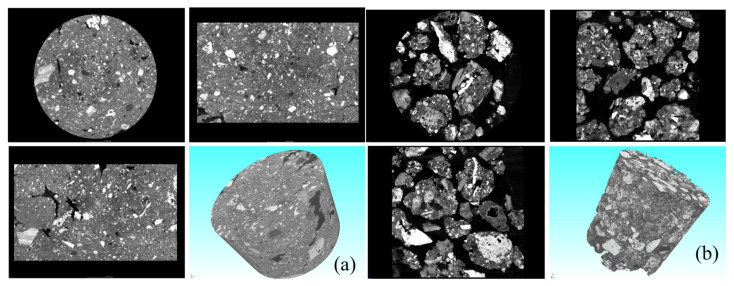
(**a**) Ordinary RM; (**b**) low-alkalinity RM CT scan quadrature view.

The mineral particle characteristics of the RM specimens before and after dealkalization were analyzed, and four views of the original RM and low-alkalinity RM were obtained using a CT scan. It can be seen from Figure 5 that the overall particles of the original RM samples have relatively small particles and high porosity, and the overall structure of the material is relatively loose due to the weak connection between the particles. After dealkalization, the low-alkalinity RM particles formed obvious aggregates, and the lower porosity made the structure more compact.

**Figure 6:**
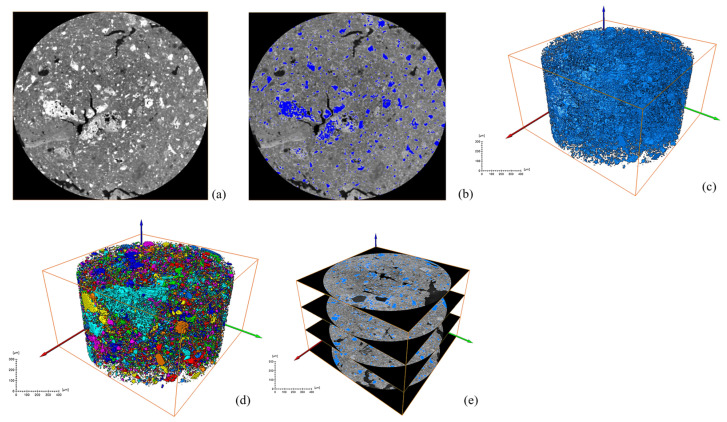
Ordinary RM: (**a**) original grayscale image; (**b**) threshold segmentation; (**c**) mineral extraction; (**d**) mineral labeling; (**e**) mineral distribution along specimen height.

**Figure 7:**
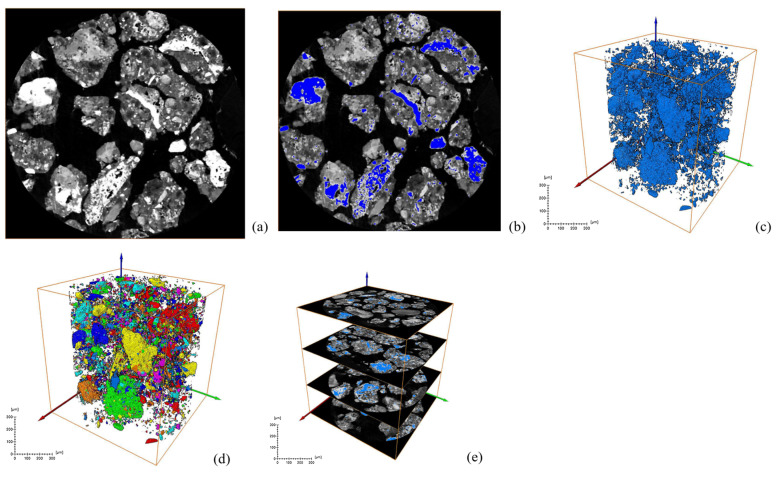
Low-alkalinity RM: (**a**) original grayscale image; (**b**) threshold segmentation; (**c**) mineral fetching; (**d**) mineral labeling; (**e**) mineral distribution along specimen height.

**Figure 8:**
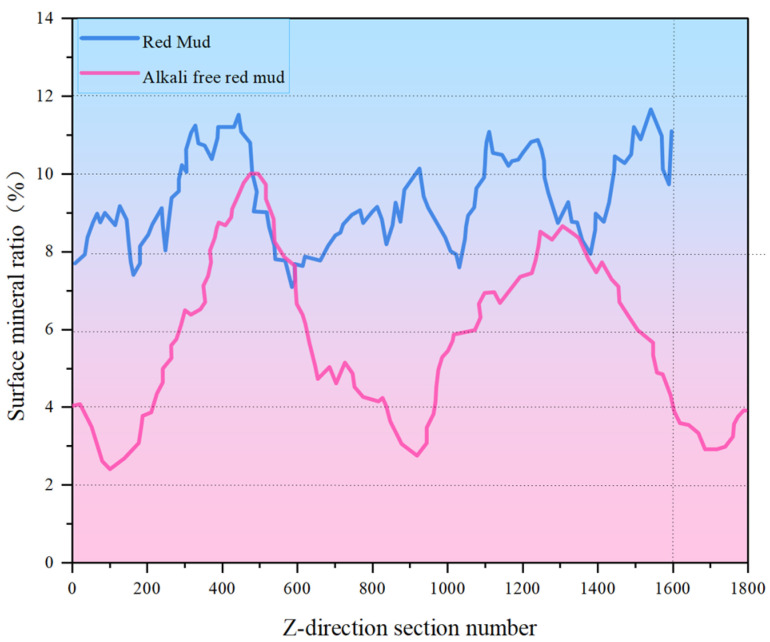
Original RM and low-alkalinity RM mineral distribution along specimen height.

After a series of slices were obtained, CT images were binarized and labeled with minerals, and the number of mineral particles in each layer was analyzed. Figure 6 and Figure 7 show the process of mineral labeling in the original RM sample and the low-alkalinity RM sample, and finally, the rate of mineral distribution along the specimen height is obtained. The results are shown in Figure 8.

Comparing Figure 6 and Figure 7, it was obvious that the particle size of RM particles becomes larger after dealkalization, and the number of small-sized minerals decreases. This is confirmed by the mineral sieve diameter distribution diagram of the original and low-alkalinity RM, which shows a significant reduction in minerals with an equivalent diameter of less than 50 μm after dealkalization. This is due to the fact that during the dealkalization process, the dissolution of CCS produces Ca^2+^ that can replace Na^+^ and Mg^2+^ in the RM. Figure 8 demonstrates the changes in the distribution of minerals along the height direction of the specimen in the original RM and the low-alkalinity RM. The mineral proportion in the original RM fluctuates greatly in the Z direction, that is, there is a large heterogeneity in the RM at the micro-scale, while the mineral distribution in the low-alkalinity RM is generally uniform. It can be seen from the comparison in the figure that the number of minerals with low-alkalinity RM at the same height is less than that of the original RM sample, which may be due to the agglomeration of small mineral particles during the dealkalization process of CCS, which reduces the number of minerals. This is consistent with the findings of Dai et al. [37], who found that Ca (OH)_2_ in CCS can react with RM to form gel products, thereby increasing the volume of mineral particles.

The changes in mineral distribution after dealkalization are mainly due to the differences in the mineral composition and physical properties of different grain sizes in RM and the effects of physicochemical changes during dealkalization. The alkali in RM mainly exists in two forms: soluble alkali and insoluble alkali [11]. NaOH and bauxite undergo a digestion reaction under high temperature and pressure, in which Al_2_O_3_ is converted into NaAlO_2_, and the resulting sodium silicate reacts under certain conditions to form hydrated sodium aluminosilicate. This insoluble alkali enters RM in the form of precipitation, and during the alkali removal process, the alkaline substances in RM react with CCS, causing changes in mineral phase and element distribution.

#### 3.1.2. Analysis of Mineral Volume and Equivalent Diameter

**Figure 9:**
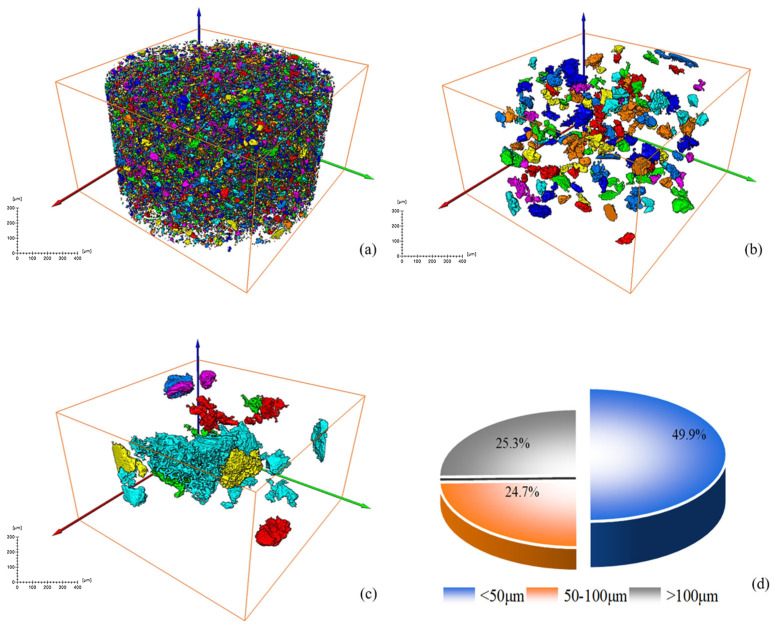
Screening and statistical analysis of minerals in CT images of original RM: (**a**) sieve diagram with equivalent diameter ≤ 50 μm; (**b**) sieve diagram with equivalent diameter 50~100 μm; (**c**) sieve diagram with equivalent diameter > 100 μm; (**d**) mineral volume percentage pie chart.

**Figure 10:**
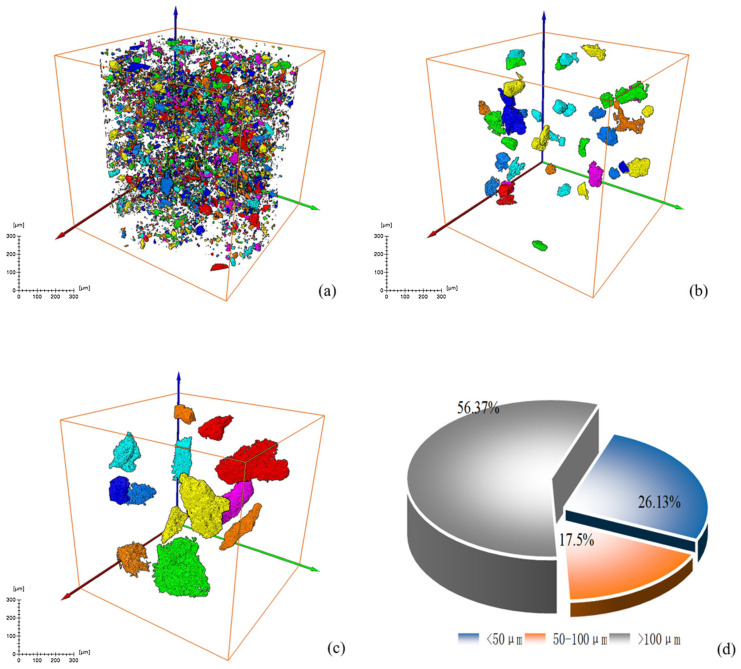
Screening and statistical analysis of minerals in CT images of low-alkalinity RM: (**a**) sieve diagram with equivalent diameter ≤ 50 μm; (**b**) sieve diagram with equivalent diameter 50~100 μm; (**c**) sieve diagram with equivalent diameter > 100 μm; (**d**) mineral volume percentage pie chart.

**Figure 11:**
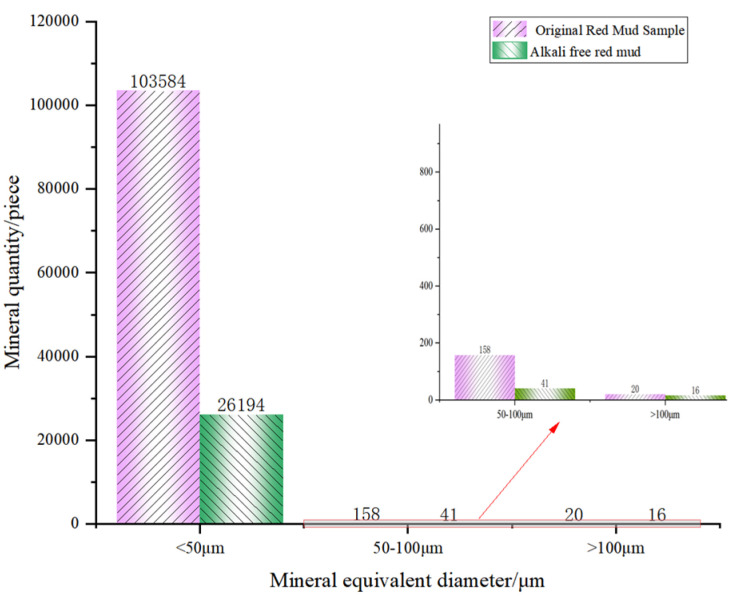
Original RM and low-alkalinity RM mineral diameter sieving statistics.

Figure 9 and Figure 10 show the volume percentage of mineral particles further sieved and obtained for different equivalent diameter minerals of the original RM and low-alkalinity RM, respectively. Figure 11 provides further statistics and supplementary information on the results shown in Figure 9 and Figure 10. It presents mineral screening statistics for different particle sizes (<50 μm, 50~100 μm, and >100 μm) of raw RM and low-alkalinity RM, providing a more comprehensive view of changes in the particle sizes of RM during the dealkalization process. After dealkylation, the mineral particles with three different equivalent particle size ranges in RM are reduced. The total number of mineral particles in the low-alkalinity RM is less than the original RM, and the mineral particles with a particle size < 50 μm decreased the most, while the mineral particles with an equivalent particle size > 100 μm decreased the least. This indicates that the dealkalization process may promote the aggregation or dissolution of particles, thus reducing the total number of particles. And the number of mineral particles with a diameter < 50 μm decreased the most in all particle size ranges, indicating that smaller particles are more likely to react in the process of dealkylation. As can be seen from the pie chart of mineral volume ratios, the volume of particles with an equivalent diameter larger than 100 μm increased greatly after the dealkalization treatment of CCS, accounting for 56.37% of the total volume, which is due to the aggregation or sedimentation of particles during the dealkalization process.

Further analysis of the calculation results shows that the percentage of minerals in the study volume (i.e., mineral rate) of the original RM is 9.32%. By analyzing the mineral percentage in the Z direction, it is found that the maximum mineral rate is about 12%, and the minimum mineral rate is 7%. The maximum mineralization rate of the low-alkalinity RM is about 10%, and the minimum mineralization rate is about 2%.

**Figure 12:**
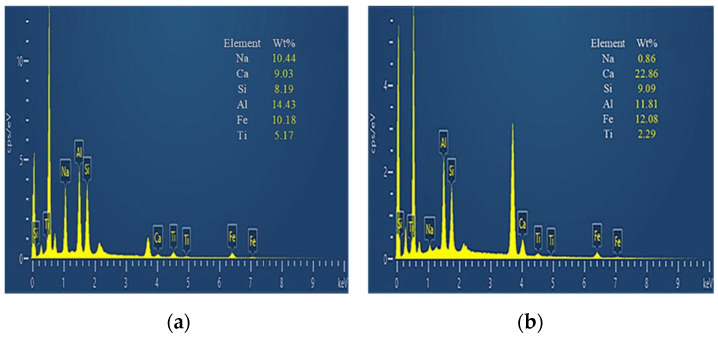
(**a**) Original RM EDS image; (**b**) low-alkalinity RM EDS image.

**Figure 13:**
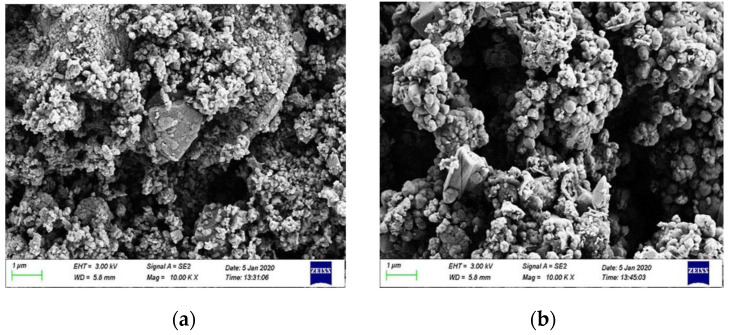
(**a**) Original RM SEM image; (**b**) low-alkalinity RM SEM image.

In general, the high porosity of the original RM and a large number of small particle minerals will reduce the strength and durability of the material, and the strong alkalinity of RM will pollute the soil and water. In contrast, the alkalinity of RM after CCS treatment is reduced, and the mineral distribution is more uniform. While the number of small particle size mineral particles is reduced, the number of large particles is increased, and the overall porosity of the material is reduced. In order to further observe the changes in the particle distribution and mineral composition of RM minerals after dealkalization, SEM-EDS analyses are carried out on the RM specimens before and after dealkalization. The results are shown in Figure 12 and Figure 13. From the EDS image, it can be seen that the Na^+^ content in RM decreased from 10.44 wt% to 0.86 wt% after dealkalization, with an alkali removal rate of approximately 91.76%, and the Ca^2+^ content increased from 9.03 wt% to 22.86 wt%, which proves that Na^+^ is replaced by Ca^2+^ in low-alkalinity RM. Compared to the dealkalization effect of traditional calcium-based materials, CCS has a good dealkalization effect on RM [38,39]. From the SEM image, it can be seen that the surface of RM before dealkalization is rough and porous with uneven distribution of elements, while the surface of low-alkalinity RM is relatively smooth and more uniform in elements. Therefore, the alkalinity of RM is reduced after CCS dealkalization treatment, and it helps to improve its performance and application value.

### 3.2. Compaction Characteristics

To boost the performance of low-alkalinity RM and meet the requirements of road use, this study mixes different solidification materials (C, CS, TG, and FA) with low-alkalinity RM to enhance the properties of materials.

**Figure 14:**
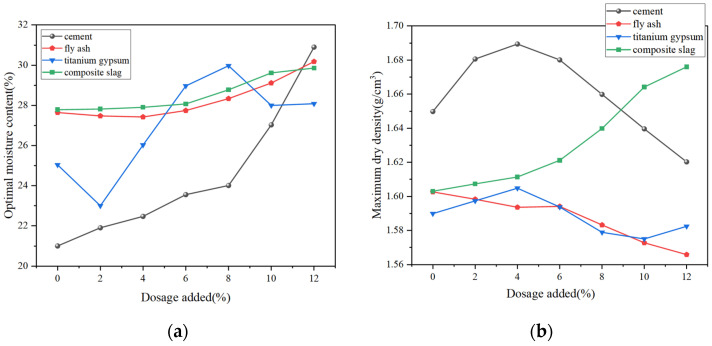
Compaction characteristics of stabilized low-alkalinity RM: (**a**) OMC values of samples with different dosages; (**b**) MDD values of samples with different dosages.

Figure 14 presents the compaction test results for stabilized low-alkalinity RM. Figure 14a,b show how different doses of four solidifiers affect the OMC and MDD values of the specimens. From the results, it can be seen that the increase in the dosage of all four stabilization materials resulted in an increase in the OMC values of the specimens, i.e., the specimens required more water to achieve good compaction. When the dosages of the four stabilization materials are increased from 0% to 12%, the OMC increases by 47.6%, 6.1%, 10.4% and 6.3%, respectively. As the content of FA and TG increases, the MDD of the specimen decreases, while the opposite trend was observed when CS was used as the stabilization material, i.e., the denseness of the specimens increased. When the dosage of the four solidifiers increases from 0% to 12%, the MDD values change by −2.12%, +5%, −0.63%, and −2.2%, respectively. Notably, C and CS have a greater impact on the material’s MDD.

As the C dosage gradually increases, the MDD of the specimen first increases and then decreases, reaching its peak at a 4% dosage. The CS replacement ratio in the range of 4% to 10% showed a strong regularity in the compaction characteristics of the specimens, while the magnitude of changes in the OMC and MDD values changed when the replacement ratio exceeded 10%. With the increase in TG as stabilization material, the OMC value decreases, increases, and then decreases again with the increase in TG dosage. The OMC value reaches the maximum when the dosage is 8%. The reason for this phenomenon can be analyzed from the particle properties of TG. It has a large specific surface area when the initial replacement ratio is low, the friction and adhesion between the particles are enhanced, and the OMC value is reduced. However, with the increase in the replacement ratio, the water absorption is enhanced, and more water is needed to achieve a good compaction effect, i.e., the physical properties of the stabilization material itself have a greater influence on the compaction effect of the specimen.

### 3.3. Mechanical Strength

#### 3.3.1. UCS

**Figure 15:**
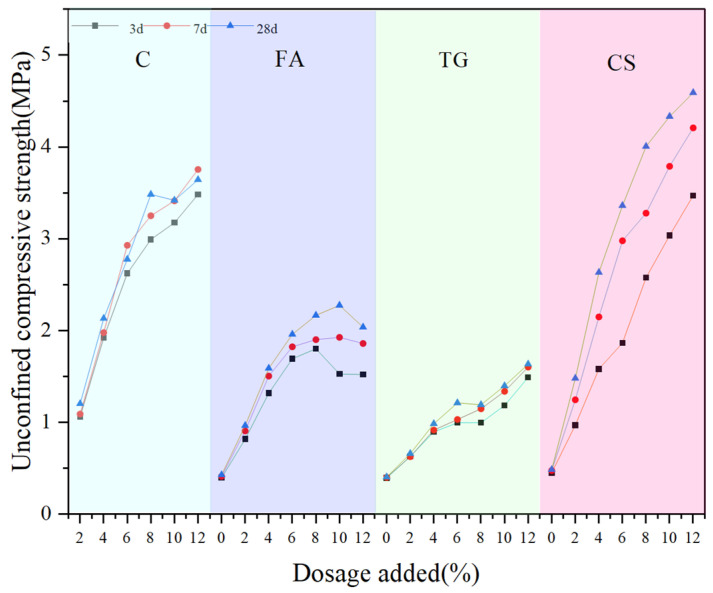
UCS values of samples with different dosages.

Figure 15 shows the UCS values of the samples at 3 d, 7 d, and 28 d under different dosages of stabilization material. It can be seen that compared to the unstabilized low-alkalinity RM material, C, CS, TG, and FA, as stabilization materials, have an obvious stabilization effect, and the UCS values of the specimens are increased to different degrees with the increase in the age of maintenance. When the curing time was 28 d, the UCS values of the specimens with 12% stabilization material, respectively, increased by 350%, 800%, 230% and 216%, compared to the control group. As the amount of cement used increases, the ucs value of the sample gradually increases, which is consistent with the research conducted by Cen et al. [22]. When C was used as stabilization material and cured for 7 d, the reaction between RM and C was almost completed, which is related to the rapid hydration reaction of cement in the early stage [40]. Therefore, the UCS values of the specimens did not change greatly with the increase in curing time.

When FA was used as a stabilization material, the strength showed a tendency to increase and then decrease at different maintenance ages, i.e., which is consistent with the results of Wang et al. [41] on the effect of FA on material stability. The maximum strength was reached at a dosage of 8%. The reason can be related to the material properties. SiO_2_ and Al_2_O_3_ in FA are two basic components of geopolymers, while RM acts as an alkali activator for the synthesis of these polymers. Therefore, it can react with RM to produce hydrated products with a certain strength [40]. Also, the fine FA particles fill the pores in low-alkalinity RM, reducing porosity and thus enhancing strength. When the dosage reaches 8%, the filling effect of excessive FA content on the internal pores of the soil reaches its limit. The additional FA particles will cause the soil structure to become looser, resulting in a decrease in the strength of the sample. Therefore, it can be seen from the figure that the UCS value decreased by 4.59% when the age of curing was 3 d and by 8% when the FA usage was 12%. After 28 d of curing, the UCS value decreased by 6.12%. Because the compounds that react with RM in the material are mainly SiO_2_ and Al_2_O_3_, the XRF analysis results of the material show that the content of both in CS is the highest, and TG is the lowest. Therefore, from the UCS test results, the magnitude of UCS values of RM stabilized by different materials at the same age and dosage is CS > C > FA > TG, i.e., CS has the best stabilization effect.

#### 3.3.2. UCS of Specimens After Immersion for One Day and Night

**Figure 16:**
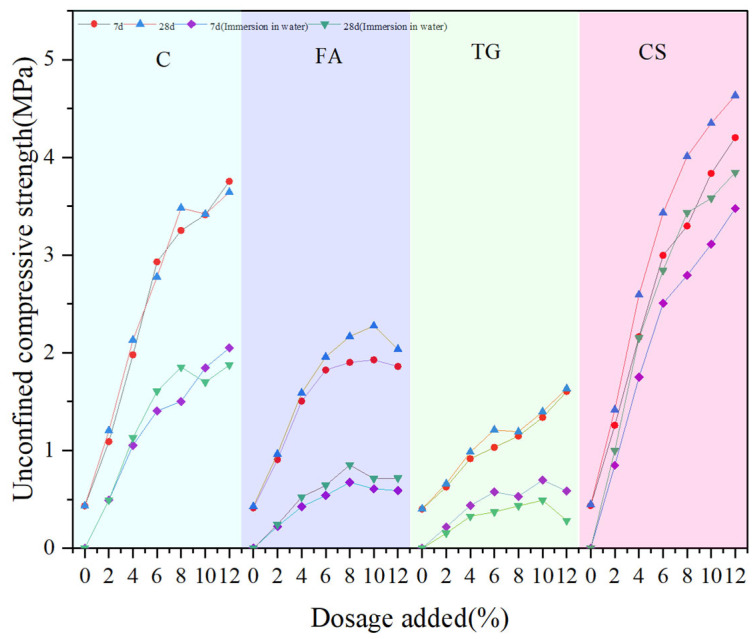
Comparison of UCS values between immersed and non-immersed specimens.

To clarify the water stability of the material, the UCS of the material under the condition of immersion for one day and night was tested and compared with that under no immersion, and the results are shown in Figure 16.

Figure 16 shows that for low-alkalinity RM stabilized materials after immersion for one day and night, the UCS values at different curing times (7 d and 28 d) are much lower than those under unsoaked conditions, but the UCS values are still influenced by the dosage of solidification materials. Water immersion leads to dissolution of soluble substances and partial hydrolysis of the C-S-H gel, weakening interfacial bonding and resulting in a lower UCS value for the material [12]. When CS is used as the stabilization material, the specimens are least affected by water immersion. At a 12% CS dosage, the UCS values at 7 d and 28 d decrease by 22.8% and 24.3%, respectively. The RM specimens doped with TG were the most affected, and the UCS value decreased by 64.7% at 7 d after immersion. And at the same curing time and dosage, the magnitude of UCS for stabilized low-alkalinity RM was CS > C > FA > TG, i.e., CS had the best stabilization effect.

**Figure 17:**
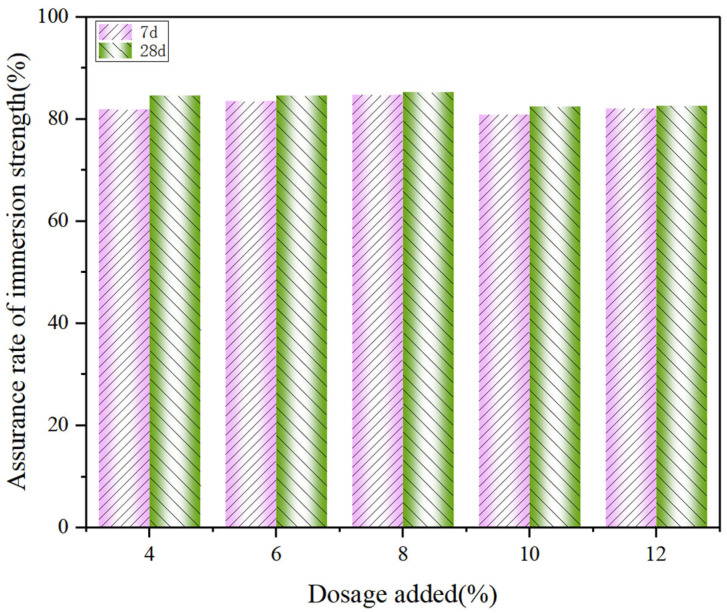
Guaranteed rate of immersion strength of low-alkalinity RM stabilized by CS.

To meet the requirements for construction and road use, the material must possess a certain level of stability. Specifically, the change in its UCS value under submerged conditions should be within a defined range. Figure 17 shows the immersion strength guarantee of CS stabilized low-alkalinity RM at the same curing time and dosage. From the results, it can be seen that the 7 d strength assurance rate of using CS to stabilize low-alkalinity RM is between 81% and 85%, and the 28 d strength guarantee rate is between 82% and 86%. It indicates that the stability effect of CS on low-alkalinity RM gradually increases with the increase in curing time. Moreover, the low-alkalinity RM materials stabilized by CS had the highest guaranteed immersion strength and the best resistance to water damage compared to other materials. It is further shown that CS is the best stabilization material for low-alkalinity RM.

### 3.4. Material Composition and Microstructure

To investigate the material composition and microstructural changes in low-alkalinity RM after stabilization, specimens were prepared using the A_4_B_3_C_4_ mix ratio from Table 1, which involves adding 8% C, 6% CS, and 8% TG to low-alkalinity RM. The specimens were then analyzed using XRD and SEM-EDX to explore the stabilization mechanisms of low-alkalinity RM.

#### 3.4.1. XRD Analysis

**Figure 18:**
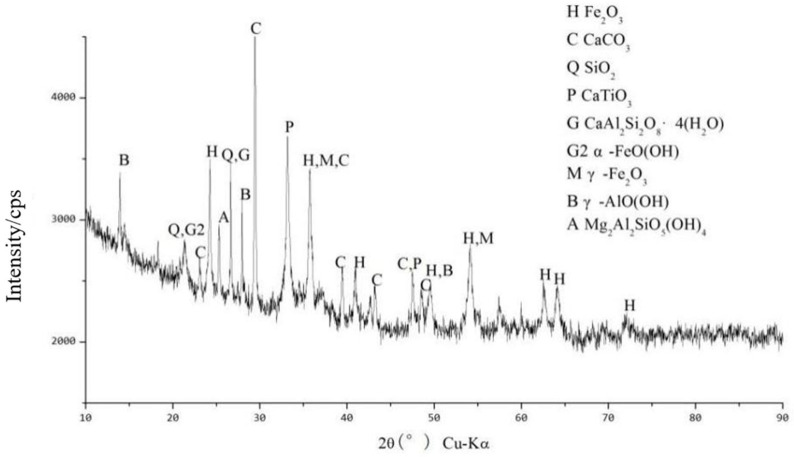
XRD pattern of low-alkalinity RM.

Figure 18 shows the XRD spectra of the original RM samples after dealkalization. From the figure, it can be seen that the main elements in the low-alkalinity RM are Fe, Al, Ca, Si, and Ti, and its main physical phases are Fe_2_O_3_, aluminum goethite, ilmenite, SiO_2_, CaTiO_3_, grossular (Ca_3_AlSi_3_O_12_), and goethite (α–FeO(OH)).

**Figure 19:**
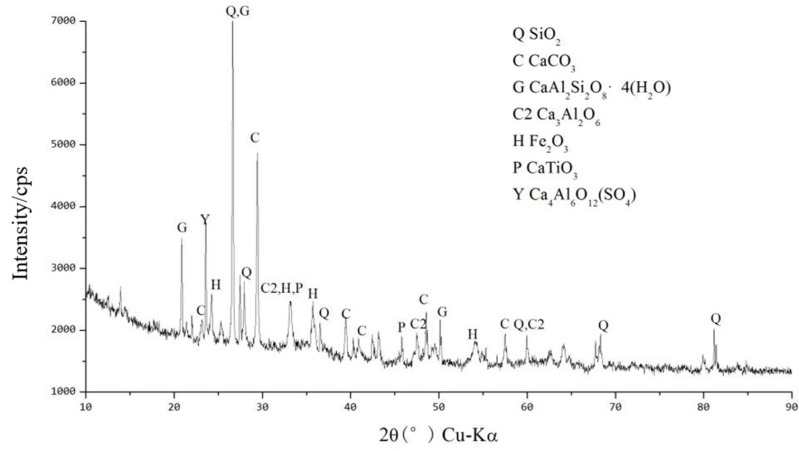
XRD pattern for stabilized low-alkalinity RM (7 d).

Figure 19 shows the XRD pattern of low-alkalinity RM after adding A_4_B_3_C_4_ and curing for 7 d. The results show that a significant stabilization reaction occurred after the addition of A_4_B_3_C_4_. The XRD pattern shows that after adding A_4_B_3_C_4_, the diffraction peaks of α-FeO(OH) and magnetite(γ-Fe_2_O_3_) disappear, while the appearance of new substances such as tricalcium aluminate (Ca_3_Al_2_O_6_) and calcium aluminate (Ca_4_Al_6_O_12_SO_4_) confirms that substances such as α-FeO(OH) are involved in the hydration reaction of the cured material with the low-alkalinity RM, resulting in new crystalline and amorphous minerals. In addition, substances such as Fe_2_O_3_, SiO_2_, and CaTiO_3_ were still present in the material, indicating that these substances may not have been involved in the reaction or the reaction was incomplete [42]. Among them, there is a high possibility that the amorphous aluminosilicate present is hydrated calcium silicate (CaOSiO_2_H_2_O, C-S-H). Such substances are usually found in the hydration reaction products of C, and given the many similarities between the reaction process and products and C-S-H, it is likely that the material will contain C-S-H [43].

#### 3.4.2. SEM-EDX Analysis

**Figure 20:**
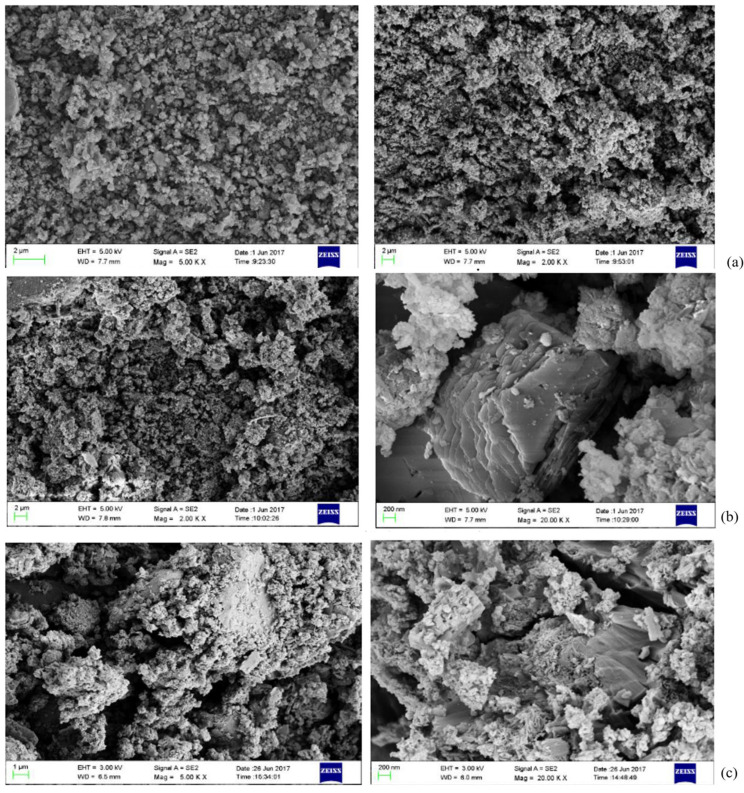
SEM images: (**a**) low-alkalinity RM; (**b**) low-alkalinity RM cured for 7 d; (**c**) low-alkalinity RM cured for 28 d.

Figure 20 shows the SEM images of low-alkalinity RM and cured low-alkalinity RM after 7 d and 28 d of maintenance. Compared with the low-alkalinity RM, the overall material structure of RM after stabilization was denser, and the number of voids was reduced. And after 28 d of maintenance, a continuous cementation layer was formed between the particles, and the stabilization reaction was more adequate.

With the addition of C, CS, and TG, the voids on the surface of the material decreased, and the plate and sheet structure increased. With the extension of curing time, the particle size of large particles increased, the average particle size was greater than 20 μm, the overall structure was more compact, and the surface was smooth. The large voids are filled by the generation of newly generated crystalline and amorphous substances, resulting in an increase in the overall strength of the RM material. The reticulated amorphous material inside the structure may be C-S-H generated by hydration, which makes the mechanical strength of the material increase. It can be seen that the crystalline prismatic and amorphous reticular materials play an important role in the curing process of low-alkalinity RM, and it is speculated that these prismatic substances might be Ca(OH)_2_ [22].

**Figure 21:**
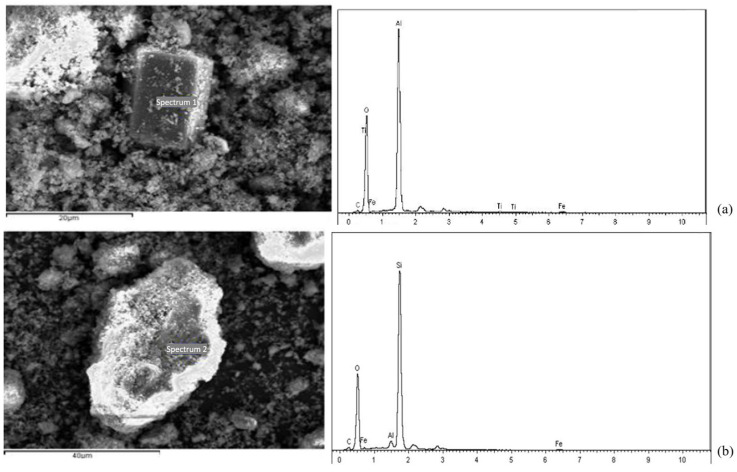
SEM images and EDX analyses: (**a**) prismatic crystalline substance; (**b**) blocky crystalline substance.

Figure 21 shows an SEM-EDX image of the typical structure inside the solidified RM material. From Figure 21a, it can be seen that the atomic ratio of aluminum element to oxygen element in the prismatic crystalline material is about 1:2, and it is basically free of common elements other than hydrogen, so it is guessed that this material may be γ-Al O(OH). In Figure 21b, it can be seen that the atomic ratio of elemental silicon to elemental oxygen in the bulk crystalline material is about 1:2, indicating the presence of SiO_2_ in the stabilized low-alkalinity RM material, and it is speculated that the lamellar structure may be a calcium-based material or tricalcium aluminate.

In summary, after adding A_4_B_3_C_4_, the Ca in the material participates in a series of hydration reactions, greatly enhancing the material’s strength. Firstly, γ-AlO(OH) and α-FeO(OH) were involved in a series of reactions to generate crystalline substances such as Ca_3_Al_2_O_6_, which was the basis for the early strength formation of RM. With the prolongation of the curing time, some amorphous states, such as reticular C-S-H, wrapped, filled, and cemented the structures with certain strength, such as CaCO_3_, Ca_3_Al_2_O_6,_ and garnet, which further increased the strength of the material.

#### 3.4.3. Analysis of Mineral Particles for Low-Alkalinity RM Stabilization Material

**Figure 22:**
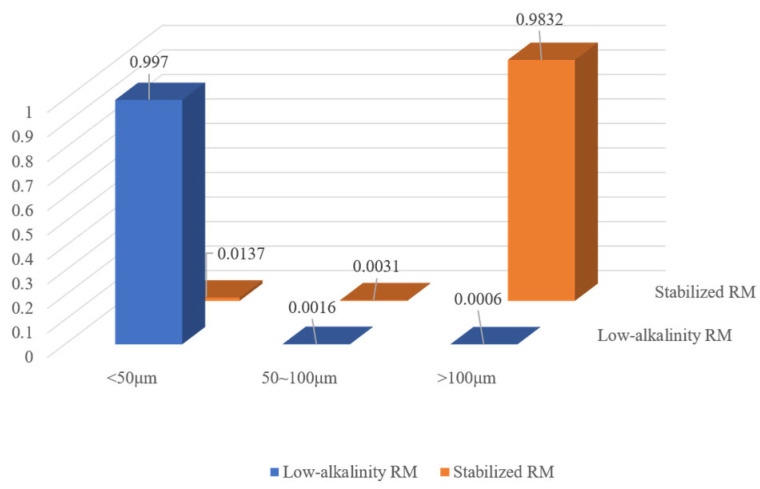
Mineral volume proportion pie chart.

The characteristics of mineral particles before and after stabilization of low-alkalinity RM were compared using a CT scan. Figure 22 shows the proportion of mineral volume in the low-alkalinity RM material after stabilization treatment. From the figure, it can be seen that the volume of mineral particles with diameters larger than 100 μm increases, accounting for 98.32% of the total volume, i.e., the material as a whole is well agglomerated, with a small number of loose particles. This is consistent with the results of SEM and EDX analysis of the material, thus further suggesting that larger agglomerates generally result in better physical stability of the material.

## 4. Summary and Conclusions

Low-alkalinity RM stabilized materials were prepared using CCS as an RM dealkalizer and C, CS, TG, and FA as stabilization materials. The study investigated the effect of CCS dealkalization on the mineral particle characteristics of RM materials and the stabilization effect of four different stabilization materials on low-alkalinity RM. Microscopic methods such as XRD and SEM-EDX were used to characterize the formation of hydration products and the stabilized mechanism during the process of low-alkalinity RM. From this study, the following conclusions can be drawn:

(1) The CT scan results showed that the particle size of mineral particles in RM after CCS dealkylation increased greatly due to the agglomeration effect, and the number of small particles decreased. The mineral distribution in RM is more uniform, and the porosity is reduced, which improves the material properties. After SEM-EDS analysis, it was found that the Na^+^ content in RM decreased from 10.44 wt% to 0.86 wt% after dealkalization, and the Ca^2+^ content increased from 9.03 wt% to 22.86 wt%. It was confirmed that Na^+^ was replaced by Ca^2+^ in RM after dealkalization, and the alkalinity was reduced.

(2) The stabilized effect of the four stabilization materials was CS > C > FA > TG. When CS dealkalized was doped with 12% and cured for 28 d, the UCS value of the specimen could reach 4.51 MPa, and the strength guarantee was between 82 and 86% after being immersed in water for one day and night. The UCS values are mainly influenced by the SiO_2_ and Al_2_O_3_ content of the material in the appropriate range, but at excessive partial replacement ratios, they are influenced by the physical properties of the doped material itself.

(3) The stabilization of low-alkalinity RM relies on crystalline and amorphous substance formation. When low-alkalinity RM is mixed and reacted with 8% C, 6% CS, and 8% TG, it generates crystalline substances like Ca_3_Al_2_O_6_, Ca_4_Al_6_O_12_SO_4_, and Ca(SiO_4_), along with amorphous substances like C-S-H. The reticular amorphous material wraps, fills, and cements the crystalline materials such as CaCO_3_ and Ca_3_Al_2_O_6_, which improves the overall strength of the material.

(4) Scanning of the stabilized low-alkalinity RM materials using CT showed a significant increase in the volume of mineral particles compared to the original RM, with mineral particles > 100 μm in diameter accounting for 98.32% of the total volume. This further suggests that an increase in the volume of mineral particles improves the properties of the material.

## 5. Prospects and Limitations

This study only investigates the performance of low-alkalinity RM stabilization materials under laboratory conditions, which differs from large-scale applications in actual situations. Future research could explore the behavior and characteristics of RM under various environmental conditions. Additionally, the synergistic utilization of RM with other solid waste materials can be explored, more efficient and economical dealkalization technologies can be developed, and the utilization rate of RM can be further improved under conditions that meet strength requirements.

## Figures and Tables

**Table 1:** Chemical compositions of RM and CCS (%).

Material	SiO_2_	Fe_2_O_3_	Al_2_O_3_	CaO	MgO	TiO_2_	Na_2_O	LOI
RM	6.88	56.59	14.82	0.52	0.18	4.61	3.11	11.26
CCS	2.82	0.20	1.80	62.60	4.20	0.08	0.06	25.4

**Table 2:** The particle size distribution of RM and CCS (μm).

Material	D10	D50	D90
RM	1.161	19.536	79.405
CCS	0.817	6.709	113.260

**Table 3:** Compound proportion in C, CS, TG, and FA (%).

Material	SiO_2_	Fe_2_O_3_	Al_2_O_3_	CaO	MgO	TiO_2_	Na_2_O	SO_2_	T_2_O_5_	LOI
C	22.26	4.38	6.64	61.24	1.12	—	—	—	—	4.36
CS	28.61	0.14	19.54	34.75	—	3.15	0.06	—	—	13.75
TG	9.45	0.04	0.18	32.14	0.40	—	—	43.48	2.18	12.13
FA	79.87			—	—	—	—	—	—	20.13

**Table 4:** Cement strength index [33].

Strength Grade	Compressive Strength/MPa		Flexural Strength/MPa	
	3 d	28 d	3 d	28 d
42.5	17.0	42.5	3.5	6.5
42.5R	22.0	42.5	4.0	6.5

**Table 5:** The particle size distribution of CS(μm).

Material	D10	D50	D90
CS	1.993	13.32	35.51

**Table 7:** Different stabilization materials and mixing ratios.

Stabilization Material	NO	Ratio	Stabilization Material	NO	Ratio	Stabilization Material	NO	Ratio	Stabilization Material	NO	Ratio
Cement	A_0_	0%	Composite slag	B_0_	0%	Titanium gypsum	C_0_	0%	Fly ash	D_0_	0%
	A_1_	2%		B_1_	2%		C_1_	2%		D_1_	2%
	A_2_	4%		B_2_	4%		C_2_	4%		D_2_	4%
	A_3_	6%		B_3_	6%		C_3_	6%		D_3_	6%
	A_4_	8%		B_4_	8%		C_4_	8%		D_4_	8%
	A_5_	10%		B_5_	10%		C_5_	10%		D_5_	10%
	A_6_	12%		B_6_	12%		C_6_	12%		D_6_	12%

**Table 6:** Physical indicators of FA.

Index	Result/%	Method [35]
0.3 mm sieve pass rate	99.02	T818
0.075 mm sieve pass rate	80.73	T818
Loss on ignition	8.10	T817
Moisture content	0.69	T801

## Data Availability

The original contributions presented in this study are included in the article. Further inquiries can be directed to the corresponding authors.
